# A preliminary study into the emergence of tendon microstructure during postnatal development

**DOI:** 10.1016/j.mbplus.2024.100142

**Published:** 2024-01-26

**Authors:** Helena Raymond-Hayling, Yinhui Lu, Tom Shearer, Karl Kadler

**Affiliations:** a Wellcome Centre for Cell Matrix Research, University of Manchester, United Kingdom; b Department of Mathematics, University of Manchester, United Kingdom; c Department of Materials, University of Manchester, United Kingdom

## Abstract

Tendons maintain mechanical function throughout postnatal development whilst undergoing significant microstructural changes. We present a study of postnatal tendon growth and characterise the major changes in collagen fibril architecture in mouse tail tendon from birth to eight weeks by analysing the geometries of cross-sectional transmission electron microscopy images. This study finds that a bimodal distribution of fibril diameters emerges from a unimodal distribution of narrow fibrils as early as the eighth day postnatal, and three distinct fibril populations are visible at around 14 days. The tendons in this study do not show evidence of precise hexagonal packing, even at birth, and the spaces between the fibrils remain constant throughout development. The fibril number in the tissue stabilises around day 28, and the fibril area fraction stabilises around day 26. This study gives coarse-grained insight into the transition periods in early tendon development.

## Highlights

• Analysed structural development of mouse tail tendons in first eight weeks postnatal
• Unimodal fibril diameter distribution becomes bimodal (day 8) then trimodal (day 14)
• Fibril number stabilises around day 26, and area fraction around day 28 postnatal

## 1 Introduction

Tendons transmit forces from muscle to bone and withstand great forces compared to other mammalian tissues. In humans, it has been reported that the forces in the Achilles tendon whilst running are as much as 9kN, or more than 10 times the average human body weight [1]. The ability of tendons to withstand these forces and avoid damaging cells within the tissue can be attributed to the structure of the tendon’s extracellular matrix. In tendon, collagen fibrils are highly aligned with the tendon’s longitudinal axis, providing tensile strength and stability. At birth, collagen fibrils are narrow (<40 nm) and of approximately uniform diameter [2], [3], though adult tendon fibrils range in diameter from 35–400 nm [4]. The adult diameter distribution has been described as both bimodal [3], [5] and trimodal [6]. How exactly the fibrils seen at birth are able to expand into the broader distribution of diameters seen at maturity whilst maintaining tendon function, is not known.

The postnatal growth of tendon tissue in mice is reported to occur in two stages [5], [7]. The first stage of tendon development (stage 1) is characterised by the presence of fibripositors. These are plasma membrane protrusions which project into the channels between cells, and are proposed to be a site of collagen fibril assembly [8]. Canty *et al*. [8] found that fibripositor formation occurs only during a narrow window of development, and they are not found in adult tendon, when the matrix architecture has been established. The second phase of tendon development (stage 2) is when fibripositors are absent, and thus *de novo* collagen synthesis is completed. During stage 2, the tissue grows and fibrils expand so that their diameters are no longer uniform. Kalson *et al*. [5] examined the structure of mouse tendon tissue at birth (during stage 1) and at six weeks. It was found that the number of fibrils in an extracellular channel (which Kalson *et al.* [5] termed ‘fibril bundles’) is unchanged between stage 1 and stage 2, and so too was the number of fibril bundles per cell nucleus. Their results, together with the disappearance of fibripositors after birth [8] suggest that the structure of tendon at birth is a template for the adult tissue, and that, although the matrix is expanding, no new fibrils are produced once the animal is born. If this is the case, then the fibrils at birth expand to have different diameters, creating a multimodal diameter distribution.

Tendon fibrils are near-circular in cross section, and do not grow indefinitely, indicating extrinsic diameter regulation. The mechanisms for fibril growth and diameter regulation are not known definitively and have been the subject of extensive research over many decades [5], [9], [10], [11], [12], [13]. However, the growth in fibril length in early development has been attributed to end-to-end fibril fusion [14], [15], and another proposed mechanism for fibril growth is the surface-nucleation-and-propagation model [16], [17], where collagen molecules attach to the tips of fibrils at specific binding sites. This discovery follows from decades of evidence that an unstable fibril nucleus forms, and subsequent molecular accretion occurs later [18], [19], [20]. Holmes *et al*. [21] observed an abrupt limitation in fibril diameter of 600 kDa/nm close to the growing, smoothly tapered tips, when fibril fragments reached a length of around 200 D-periods. These results suggest that the limit of a fibril’s diameter relies on its surface structure.

Tendons maintain their mechanical function through development, despite undergoing dramatic microstructural changes. To understand how they do this, it is of interest to uncover when and how the distribution of tendon fibrils shifts from that characteristic of developmental stage 1 (narrow fibrils of uniform diameter) to that of stage 2 (multimodal diameter distribution). A detailed understanding of this developmental phenomenon may give crucial insight into the mechanism that is responsible for matrix expansion and fibril diameter regulation.

This study uses transmission electron microscopy (TEM) together with computational techniques to identify and characterise the emergence of the multimodal distribution of collagen fibril diameters. By investigating the window of time where tendon tissue switches from stage 1 to stage 2, the aim of this work was to trace and quantify the structural changes that occur within tendon throughout the first eight weeks postnatal, using TEM images to provide insight into matrix expansion in tendon tissue. Quantitative results are presented relating to: the fibril diameters, the form of the fibril diameter distribution, the geometric arrangement of the fibrils in the intercellular space, the proximity of the fibrils to each other, the fibril number density and the fibril area fraction.

## 2 Methods

### 2.1 Animal work

Sample dissection and imaging were conducted at the University of Manchester. The care and use of all mice in this study were carried out in accordance with the UK Home Office regulations, UK Animals (Scientific Procedures) Act of 1986 under the Home Office Licence (#70/8858 or I045CA465). Four timed matings of C57BL/6 mice were set up to collect tail tendons from the day of birth (day 1), daily up to age 10 days, then every 4–5 days until day 35 (5 weeks) then weekly until day 56 (8 weeks). Mice in the litter were sacrificed at the same time each day (12:00). At time points until day 7, a section of the tail was dissected from the central portion with a cross-sectional cut. After day 7, the tail section was further bisected longitudinally to ensure sufficient penetration of the decalcification buffer and fixative. After day 10, the tails were excised, and a tendon segment was extracted from the proximal tail tendons of each mouse. One mouse was used at each time point. The mice were of mixed sex, so as not to introduce sex-based bias, and from two litters born two days apart. This decision was made to minimise animal sacrifice. This was considered appropriate due to the genetically identical nature of the C57BL/6 strain.

### 2.2 Transmission electron microscopy (TEM)

Tendons were prepared for transmission electron microscopy (TEM) as described in [22]. In brief, tendons were fixed in 1% osmium tetroxide (w/v) and 1.5% potassium ferrocyanide (w/v) in 100 mM sodium cacodylate buffer (pH 7.2) for 2 h, washed with double distilled water (3 x 5 min), then incubated with 1% tannic acid (w/v) in 100 mM cacodylate buffer for 1 h, washed with double distilled water, then placed in 1% osmium tetroxide (w/v) in water for 30 min. Samples were then washed with distilled water and stained with 1% uranyl acetate (aqueous) for 1 h, then dehydrated in graded ethanol (30%, 50%, 70%, 90% for 8 min at each step then 4 x 8 min each in 100% ethanol), and transferred to acetone for 10 min. The samples were then passed through a graded series of Agar100Hard in acetone and allowed to cure at 60°C for 72 h. Sections (60 nm) were cut and examined using a Tecnai 12 BioTwin electron microscope.

Between 5 and 27 TEM images were analysed at each time point. Images unsuitable for automatic segmentation (those with low contrast due to poor stain penetration), or with many artefacts due to damage incurred during sectioning, were discarded. The images were collected from different sections of multiple tail tendons to maximise coverage across the tail. The numbers of images, and the ultimate numbers of fibrils analysed at each time point are detailed in Table 1.

**Table 1:** Number of images and fibrils analysed at each time point in the study.

**Day**	**Images**	**Fibrils**	**Day**	**Images**	**Fibrils**
1	9	2076	14	22	14015
2	6	1794	16	20	11887
3	12	2309	18	21	9362
4	11	2852	21	19	13544
5	11	2906	25	22	7588
6	8	1973	28	27	6633
8	11	3018	30	17	7121
9	15	3725	35	21	5798
10	5	1422	42	23	6230

### 2.3 Segmentation and analysis

**Fig. 1:**
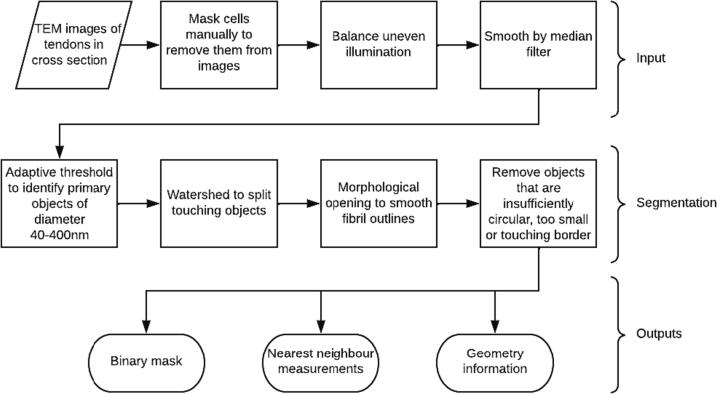
Summary of CellProfiler pipeline. The outputs of this process yield useful structural information for comparison of tendon morphology throughout early postnatal development.

To understand the evolution of tendon morphology and architecture, it was necessary to characterise changes in fibril arrangement and shape quantitatively. Several fibril shape parameters and their centroids were measured. To do so, a pipeline for segmentation and quantification was designed using CellProfiler. CellProfiler is an open-source program which combines standard Python modules and other computer vision packages [23]. A summary of the pipeline is outlined in Fig. 1. Starting with the images obtained by TEM, cells were removed from the image by manual tracing to produce a mask. The CellProfiler pipeline was then used to identify fibrils and separate them from the interfibrillar spaces and nearby tendon cells. The pipeline uses an adaptive threshold to identify fibril segments and seperate them from the background. In this technique, the input image is divided into tiles and a threshold is recalculated for each tile to allow for uneven stain or illumination. For older tendons, an additional watershedding step is introduced, which is a means of splitting apart fibrils which are touching. Morphological opening is used to smooth the outlines of the fibrils, and objects which are too small, or of a highly irregular shape are removed, as well as those touching the border of the image. The border fibrils were put in a separate class, to be included when calculating the fibril area fraction, but excluded from other measurements. The parameters were adjusted slightly through the time series to account for changes in image magnification, increasing fibril size and decreasing relative interfibrillar gap size, though the key steps in the pipeline remained unchanged.

Many of the images contained large regions occupied by cells, which made them unsuitable for some of the later analysis on fibril packing and geometrical arrangement (see Section 3.5). For these tasks, it was essential to focus on the intracellular spaces, so an image with no cells was selected. Thus, the results in Fig. 8, Fig. 9, Fig. 10 are calculated from one image per day. All other analysis used all images (see Table 1 for numbers).

## 3 Results

### 3.1 TEM images

One image of the embedded section of tendon collected for each time point is displayed in Table 2. The fibrils are of uniform diameter in the first few days postnatal, and the emergence of the multimodal distribution of fibrils evolves slowly until it is it clearly visible around day 21. To understand the emergence of this diameter distribution quantitatively, the segmentation and measurement pipeline outlined above was applied to these images.

**Fig. 2:**
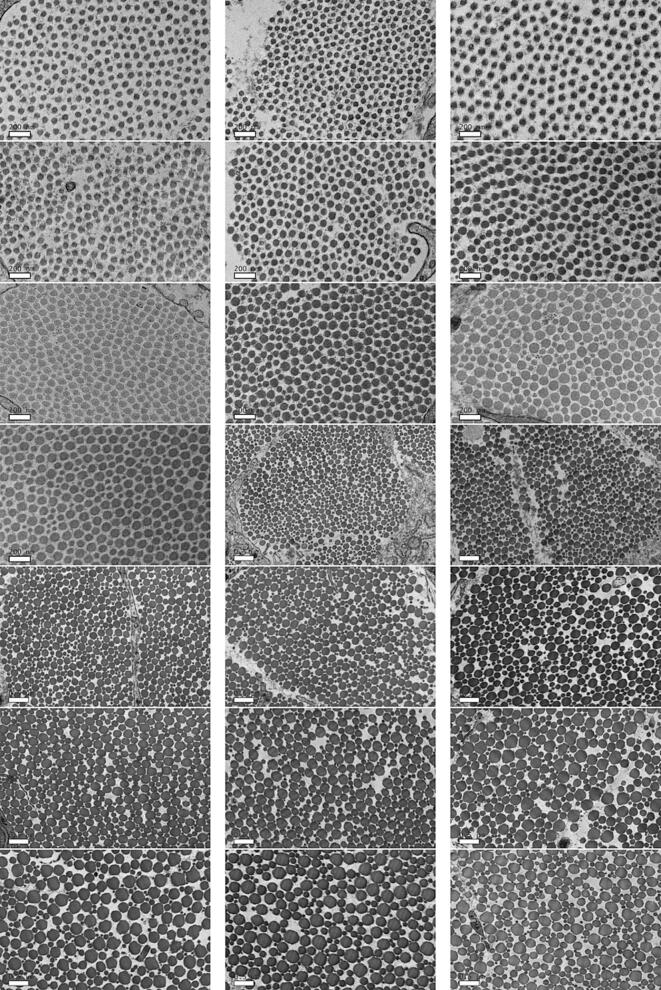
TEM images of mouse tail tendon in the first six weeks of life. From top left to bottom right, days postnatal: 1, 2, 3, 4, 5, 6, 7, 8, 9, 10, 14, 16, 18, 21, 25, 28, 30, 35, 42, 49, 56. The scale bars on days 1–10 are 200 nm, and on all other days are 500 nm.

### 3.2 Image segmentation

**Fig. 3:**
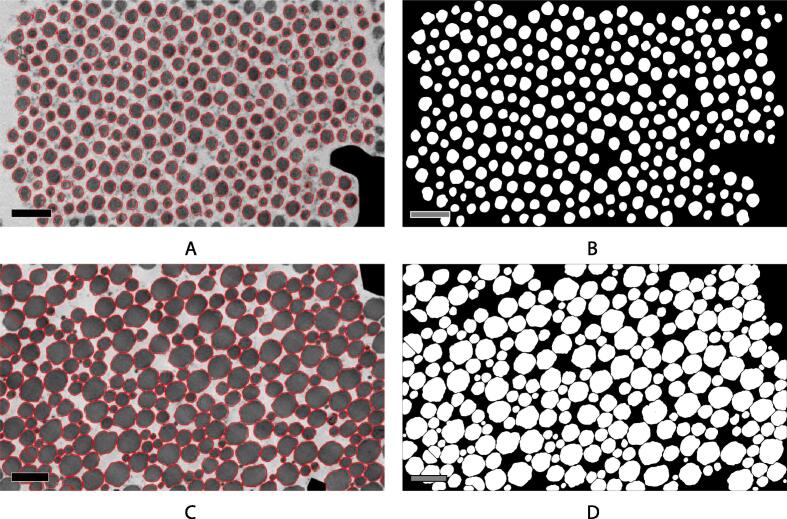
Typical segmentation achieved with the CellProfiler segmentation pipeline. (a) Fibrils at an early stage of development (day 5) overlaid with their outlines as detected by the custom built CellProfiler pipeline, with the corresponding binarised fibril mask (b). Scale bars in (a) and (b) are 200 nm. (c) Fibrils at a later stage of development (day 35) overlaid with their outlines as detected by the custom built CellProfiler pipeline, with the corresponding binarised fibril mask (d). Scale bars in (c) and (d) are 500 nm. Here, the segmentation requires a watershedding step to separate fibrils which are touching. The high level of accuracy across the developmental series shows the efficacy and flexibility of the CellProfiler pipeline for detecting fibrils with different morphologies and arrangements.

The segmentation of fibrils using the CellProfiler method is highly successful for fibrils in both early development, which are narrower and spaced further apart, and later, when the fibrils are of more variable size and are separated by just a few pixels. Some typical results of the segmentation are given in Fig. 3, showing the accuracy of this method. The morphology and arrangement of the fibrils were quantified in the CellProfiler analysis and were used to examine a range of structural quantities through the time series.

### 3.3 Capturing the emergence of the multimodal fibril distribution

**Fig. 4:**
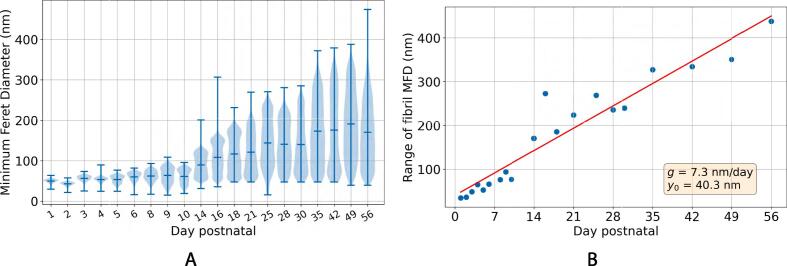
Overall trends in Minimum Feret diameter (MFD) distribution in the first eight weeks postnatal, calculated using all fibrils in all images (see Table 1). (a) Violin plot illustrating the shape of the MFD distribution throughout the time series. The horizontal line in the middle of the violin plot shows the mean of the data, and the bars at the top and bottom represent the maximum and minimum values. The MFD distribution starts off narrow and centred around smaller values, before gradually broadening and displaying multiple modes as the tendon grows. The horizontal axis is spaced according to the time series data points and is thus non-linear. (b) The range of MFDs at each time point. This plot illustrates the broadening of the distribution throughout .the time series.

The first avenue of investigation relates to the shape of the distribution of the fibrils’ minimum Feret diameters (MFDs) and how it evolves over time. A violin plot showing the distribution of MFDs is given in Fig. 4A. The evolution of the distribution is clear here. At early time points, the fibrils are narrower, the range of MFDs is narrow, and there is one clear peak in the distribution. As time goes on, multiple fibril populations are visible, and both the mean MFD and the range of values increases. The range of MFDs is plotted in Fig. 4B, and appears to grow linearly. To analyse the distributions in Fig. 4A, the data were fitted with normal, bimodal and trimodal distributions to inspect how many populations of fibrils are present at each stage of development.

The dataset at each time point was fitted firstly with a standard normal distribution:

(1) $$N\left(\mu ,\sigma^{2}\right)=\frac{1}{\sigma \sqrt{2\pi }}e^{-\frac{1}{2}{\left(\frac{x-\mu }{\sigma }\right)}^{2}},$$

where the mean and variance of the distribution are μ and $\sigma^{2}$ respectively, and *x* is the MFD of a fibril. We then fit a bimodal distribution, a sum of two weighted normal distributions with means $\mu_{1}$ and $\mu_{2}$, and variances $\sigma_{1}^{2}$ and $\sigma_{2}^{2}$, and weights *w* and 1-w (where 0<w<1), such that its probability density function (PDF) is given by

(2) $$P_{b}\left(\mu_{1},\sigma_{1}^{2},\mu_{2},\sigma_{2}^{2},w\right)=wN\left(\mu_{1},\sigma_{2}^{2}\right)+(1-w)N\left(\mu_{2},\sigma_{2}^{2}\right)\text{.}$$

Similarly, we fitted a trimodal distribution, a sum of three weighted normal distributions with means $\mu_{1},\mu_{3}$ and $\mu_{3}$, variances $\sigma_{1}^{2},\sigma_{2}^{2}$ and $\sigma_{3}^{2}$, and weights $w_{1},w_{2}$ and $1-w_{1}-w_{2}$ (where $0<w_{1},w_{2}<1$), with PDF

(3) $$P_{t}\left(\mu_{1},\sigma_{1}^{2},\mu_{2},\sigma_{2}^{2},\mu_{3},\sigma_{3}^{2},w_{1},w_{2}\right)=w_{1}N\left(\mu_{1},\sigma_{2}^{2}\right)+w_{2}N\left(\mu_{2},\sigma_{2}^{2}\right)+(1-w_{1}-w_{2})N\left(\mu_{3},\sigma_{3}^{2}\right)\text{.}$$

**Fig. 5:**
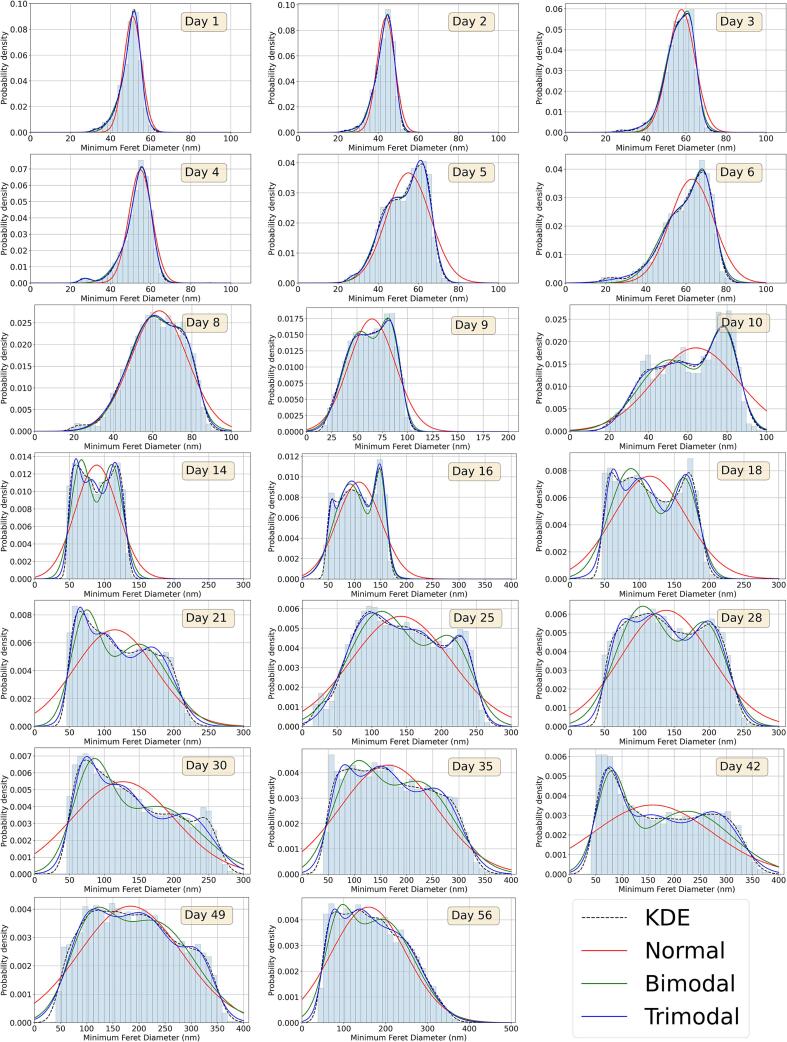
MFD distributions of mouse tail tendon fibrils in the first six weeks of life. At each time point, three different functions are fitted to the data. The normal distributions are displayed as red lines, the bimodal distributions as green lines, and the trimodal distributions as blue lines. A kernel density estimate (KDE) for each is given as a dashed black line.

**Fig. 6:**
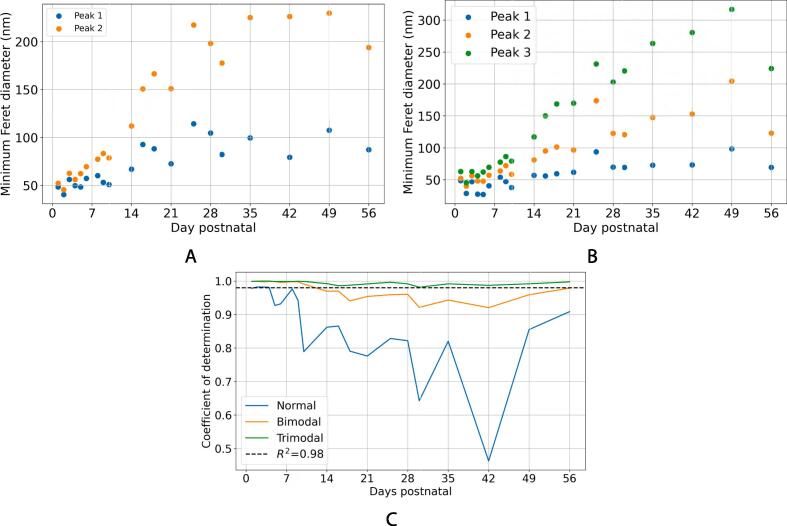
Locations of peaks for bimodal (a) and trimodal (b) fits to MFD distributions (given in Fig. 5) in the first eight weeks postnatal. Two distinct peaks in the data are visible around day 8, and three are apparent around day 14. (c) Coefficient of determination ($R^{2}$) for normal, bimodal and trimodal distributions over time. The dashed line indicates the val.ue $R^{2}=0.98$.

The distribution of MFDs across the time series, overlaid with the three fitted PDFs is given in Fig. 5. Of course, fitting more peaks to this data will only give a closer fit, so the quality of fit is not the only important factor in considering the nature of the MFD distribution. How distinct the peaks are is also worth attention. To visualise this, the peaks of the bimodal and trimodal fits are plotted in Fig. 6. In the bimodal fits (6A), the peaks are close together until around day 8, when two distinct populations emerge, and the separation of the peak location continues to increase. This suggests that a second fibril population appears around day 8. Before this time, the fibrils are all of a similar size. Turning to the trimodal fits (Fig. 6B), the three peaks are close together until day 14, when they separate. This data suggests that day 14 marks the onset of the trimodal distribution, which continues to develop as the fibrils grow. In Fig. 6C, we plot the coefficient of determination ($R^{2}$) for the normal, bimodal and trimodal fits (compared with the KDE from Fig. 5). It can be seen that the quality of fit for the normal and bimodal fits decreases over time as the modality of the data increases. The value of $R^{2}$ remains below a threshold of 0.98 after the days identified as the transitions between uni- and bi-modal (day 8) and bi- and tri-modal (day 14) data. It must be noted that the number of peaks in the distribution is arbitrary, so the assumption of trimodality may be an oversimplification.

### 3.4 Matrix expansion: fibril area fraction and fibril number density

**Fig. 7:**
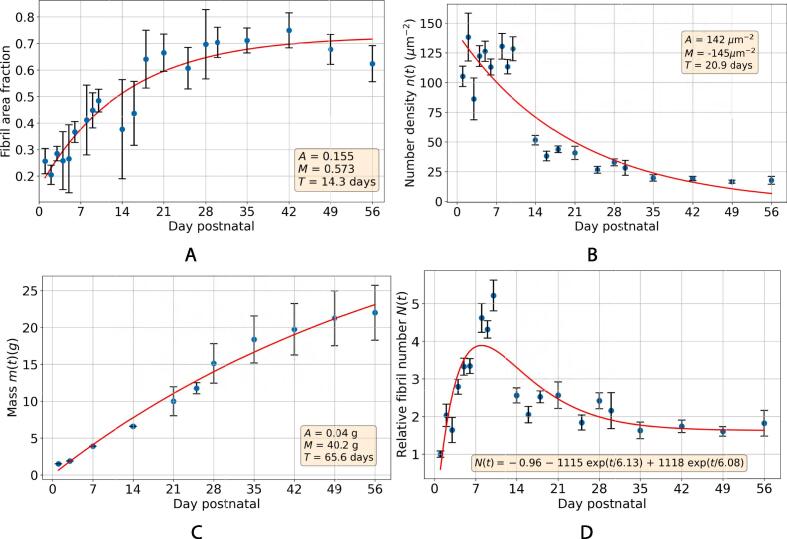
(a) FAF in the first eight weeks postnatal. (b) Number of fibrils per square micron in the first eight weeks postnatal. (c) Growth curve showing the mass of a C56/BL6 mouse (mean value of male and female) over time, using results from [24], [25]. (d) Relative fibril number (calculated in terms of the number at day 0). This quantity is calculated using the fibril number density in (b) and the animal growth curve in (c) through Eq. (8), to account for animal growth as well as fibril growth. The bars indicate standard deviations. For (a), (b) and (d), these were calculated using all fibrils in all images (see Table 1).

As discussed, for the microstructure to evolve from a population of narrow fibrils of uniform size to larger fibrils with a multimodal distribution of diameters, either new, narrow fibrils need to be synthesised postnatally, or the narrow fibrils seen in the first few days postnatal must grow to different sizes. To investigate this question, the fibril area fraction (FAF) and fibril number density were calculated. The FAF (ϕ) was calculated as

(4) $$\phi =\frac{A_{f}}{A_{T}-A_{c}},$$

where $A_{f}$ is the area occupied by the fibrils, $A_{T}$ is the area of the whole image, and $A_{c}$ is the area occupied by the masked cells. The FAF was calculated for each image at each time point, as displayed in Fig. 7A. It increases rapidly in the first few days postnatal, plateauing at a value around 70%–75% (with a possible slight decrease between days 42 and 56). It is expected that when the fibril number density is no longer changing, no new fibrils are being produced. Fibril number density, n(t) (μm^−2^), is plotted in Fig. 7B. It decreases rapidly after birth before plateauing at a value around 20 μm^−2^.

Using the same sized area to calculate fibril number density per unit area will not give an accurate indication of the number of fibrils per tendon, as the tendon (and the animal) is growing throughout this period. To account for this, a growth curve for a typical C57/BL6 mouse was considered (see Fig. 7C). This curve is calculated using the mean value of male and female mice, which reflects the mixed population used in this study (see Section 2.1). This is used to approximate a quantity that is proportional to the number of fibrils per tendon, N(T). To do this, it is supposed that the lateral expansion of the tendon fibrils in the 2D images is related to the lateral expansion of the tissue as a whole. The growth curve given in Fig. 7C is given in terms of mass, which, assuming that mice maintain a constant mass density (which is likely close to that of water) throughout their growth, is proportional to volume. As volume scales with $L^{3}$, where *L* is a length measurement, and area with $L^{2}$, the expected lateral expansion of the cross-sectional area, A(t), of a mouse’s tissues can be approximated using

(5) $$A(t)\propto m{\left(t\right)}^{2/3},$$

where m(t) is the growth curve (the mass of the animal over time). The unknown proportionality constant accounts for the exact geometry of the animal, the shape of the tendon tissue in cross-section, and the animal mass density. Using Eq. (5), an approximation of the number of fibrils over time, called the relative fibril number N(t), can be calculated:

(6) $$N(t)\propto n(t)m{\left(t\right)}^{2/3}\text{.}$$

As the constant of proportionality is not known, the relative fibril number, is normalised so that it is equal to 1 at t=0. The relative fibril number is plotted in Fig. 7D. We observe a rapid increase in this quantity until approximately day 10 after birth, before a decrease to a final value just less than twice the value at day 1. This may indicate that a large number of fibrils are laid down in the first ten days postnatal, but that many of these merge to form larger fibrils in the following days.

In Fig. 7A–C, an exponentially decaying function f(t) was used to fit the data,

(7) $$f(t)=A+M(1-e^{-t/T}),$$

where A,M and *T* are the fitting constants. In Fig. 7D, the following function was used to fit the data:

(8) $$g(t)=A+M_{1}e^{-t/T_{1}}+M_{2}e^{-t/T_{2}},$$

where $A,M_{1},M_{2},T_{1}$ and $T_{2}$ are the fitting constants.

### 3.5 Geometric packing of fibrils

**Fig. 8:**
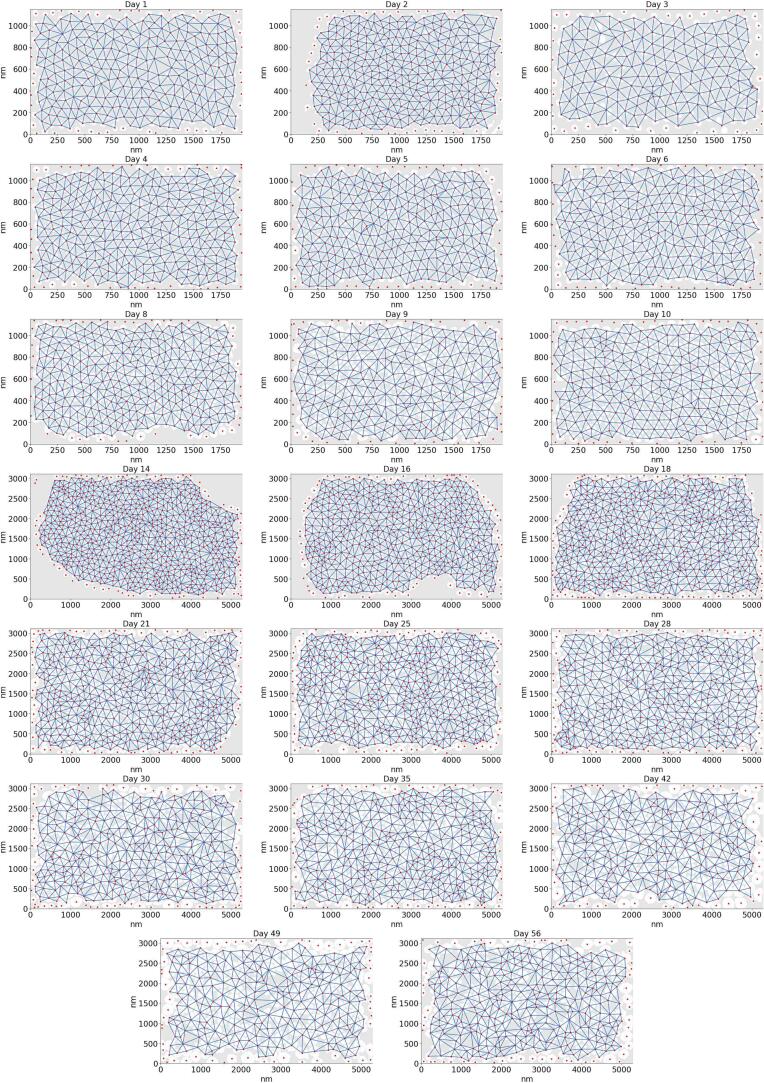
Delaunay triangulation of fibrils in TEM images of murine tail tendon in cross-section in the first eight weeks of life. Red markers represent fibril centroids, as detected by CellProfiler segmentation, and blue lines represent the edges which connect these vertices. Edges connected to the vertices at the periphery (lying on the convex hull) are excluded, as inaccurate edges are drawn by the Delaunay triangulation along the perimeter.

With regard to the geometrical arrangement of the fibrils, an image for each time point was selected that contained no cells, and a Delaunay triangulation of the centroids of all the fibrils in each image was computed. This produces a graph, with the centroid of each fibril as the vertices, and the lines connecting nearest neighbours as the edges. Next, a concave hull (alpha shape) was calculated for these vertices and all edges connected to vertices lying along the perimeter of the alpha shape were removed. This is because these outer vertices were connected to each other in error by artificially long edge lengths. The Delaunay triangulation for each image is given in Fig. 8, overlaid on the binarised image, with markers representing the fibril centroids.

With this triangulation complete, it is possible to analyse the geometric arrangement of the fibrils quantitatively. First, the angles in each three-vertex triangle in the graph were computed. This is all groups of three vertices in which all three vertices are adjacent to each other. In a perfect hexagonal array, all of these angles would be 60°. How tightly the angles are distributed around 60°gives an indication of how close to a hexagonal array the Delaunay triangulation is. It is interesting to note that, though the fibrils might appear to be arranged more regularly at the early time points in the images (see images in Table 2), the angles in the graph are not noticeably closer to a uniform 60°at day 1 than at day 56. Though the fibrils in the images acquired here are close to uniform in size in the first few days postnatal, their geometric arrangement cannot be described as hexagonal or regular.

**Fig. 9:**
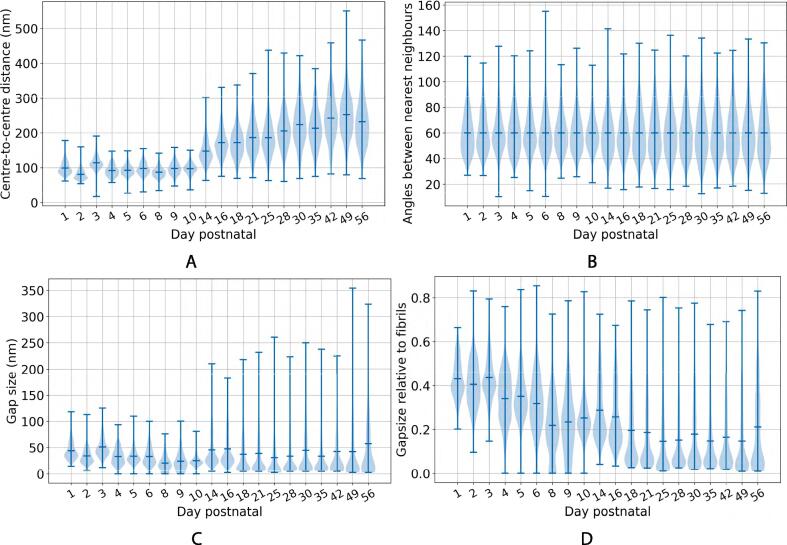
Quantification of fibril geometry, calculated using Delaunay triangulation, throughout the time series. In all plots, the horizontal axis is spaced according to the time series data points and is thus non-linear, the horizontal lines in the middle of the violin plots show the means of the data, and the bars at the top and bottom represent the maximum and minimum values. (a) The centre-to-centre distance between the fibrils and their nearest neighbours. (b) The angle between fibril centroid nearest neighbours. (c) The interfibrillar gap size, along the edge, connecting adjacent fibril centroids. (d) The interfibrillar gap size as a fraction of the mean MFD of the fibrils either side of the gap.

In Fig. 9A, the edge lengths of the triangulation are shown, which represent the centre-to-centre distances of nearest fibril neighbours. The mean edge length increases and the distribution of edge lengths becomes more widely spread as the mouse grows. The edge lengths in this graph are a combination of both the radii of the fibrils and the space between them. Separately given is the distribution of interfibrillar gap sizes (in the direction joining adjacent centroids) over time and the ratio of these gap sizes to the mean MFDs of the fibrils either side of the gap (Fig. 9C - D). Surprisingly, the mean size of the gaps between fibrils does not appear to increase with time, staying around 50 nm. The fibrils appear closer together in the images as they themselves are growing, and the gap size decreases relative to the average size of the fibrils.

**Fig. 10:**
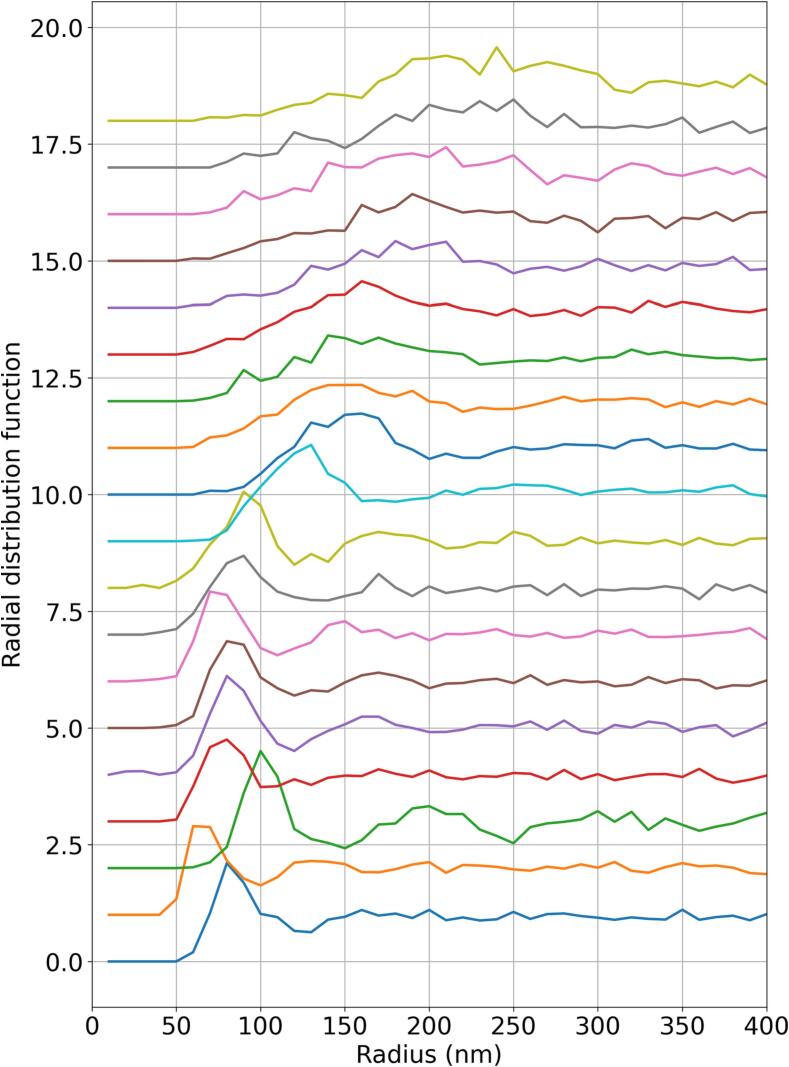
RDF of fibrils for each day. Each curve is offset vertically from the previous one by 1, for clarity. Day 1 has 0 offset. The lines from bottom to top are days: 1, 2, 3, 4, 5, 6, 8, 9, 10, 14, 16, 18, 21, 25, 28, 30, 35, 42, 49, 56.

In Fig. 10, the radial distribution function (RDF) is plotted for each day, which gives the number of fibril centres in disks with thickness *dr* and variable radii, as plotted on the horizontal axis. We used dr=10 nm to generate these results. The RDF gives well-defined peaks at regular intervals for crystalline and quasi-crystalline materials. We found slight evidence of a quasi-crystalline structure for the first few days postnatal, especially on day 3; however, in general, the structure appears to be amorphous. Meek and Knupp [26] used the RDF to analyse the spatial distribution of collagen fibrils in the cornea and found much more pronounced regular peaks, indicating a quasi-crystalline fibril arrangement. This may be due to the requirement to allow light to pass through the cornea unimpeded. Tendons have no such requirement, which may explain their relatively amorphous structure. The location of the first peak in the RDF moves rightwards and becomes less pronounced with time. This indicates that the average fibril centre-to-centre distance increases, and that the initially ordered fibril arrangement becomes more disordered, as the tendon grows.

### 3.6 Fibril shape

**Fig. 11:**
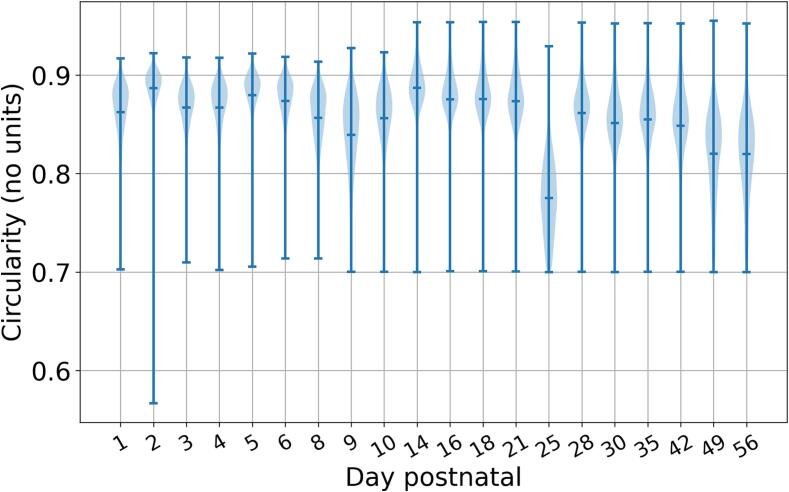
The circularity of the fibril populations throughout the first eight weeks postnatal, calculated via Eq. 9. The horizontal axis is spaced according to the time series data points and is thus non-linear. The horizontal lines in the middle of the violin plots show the means of the data, and the bars at the top and bottom represent the maximum and. minimum values.

The shape of the fibrils was also of interest in this investigation. If fibrils are highly irregular in shape (non-circular), this could give evidence of lateral fibril fusion events. To identify non-circular fibrils, the circularity *C* was measured for each fibril in each image, which is given by

(9) $$C=\frac{4\pi A}{P^{2}},$$

where *A* is the fibril area and *P* is the fibril perimeter. For a perfect circle, C=1. The circularity is plotted for the time series in Fig. 11. Fibril circularity appears to decrease slightly from the early time points through to maturity. This supports the hypothesis that some lateral fusion does occur.

### 3.7 Presence of fibripositors

**Fig. 12:**
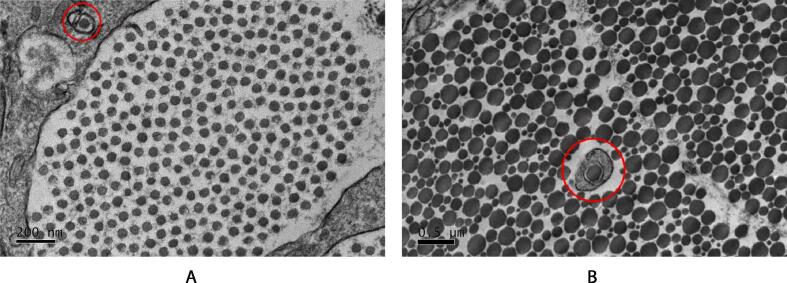
Fibripositors observed in the developmental series. (a) Fibripositors are seen in early development. This image is of a tendon cross section on the second day postnatal (day 2). Though rare, we observed the presence of fibripositors as late as day 25. (b) The retraction of fibripositors marks the end of developmental stage 1. Fibripositors are indicated by red circles.

Though no precise quantification of the number of fibripositors over time was conducted in this work, the existence of fibripositors *was* observed in the images captured. At early time points, fibripositors are expected, and were seen in the first days postnatally (Fig. 12A), they were also observed as late as day 25, when the multimodal distribution had already become apparent (Fig. 12B). This is interesting as it has been previously suggested that fibripositors disappear early on in postnatal development, and *de novo* collagen fibril formation has ceased. Though rare, the presence of fibripositors so late in tendon development is curious.

## 4 Discussion

**Fig. 13:**
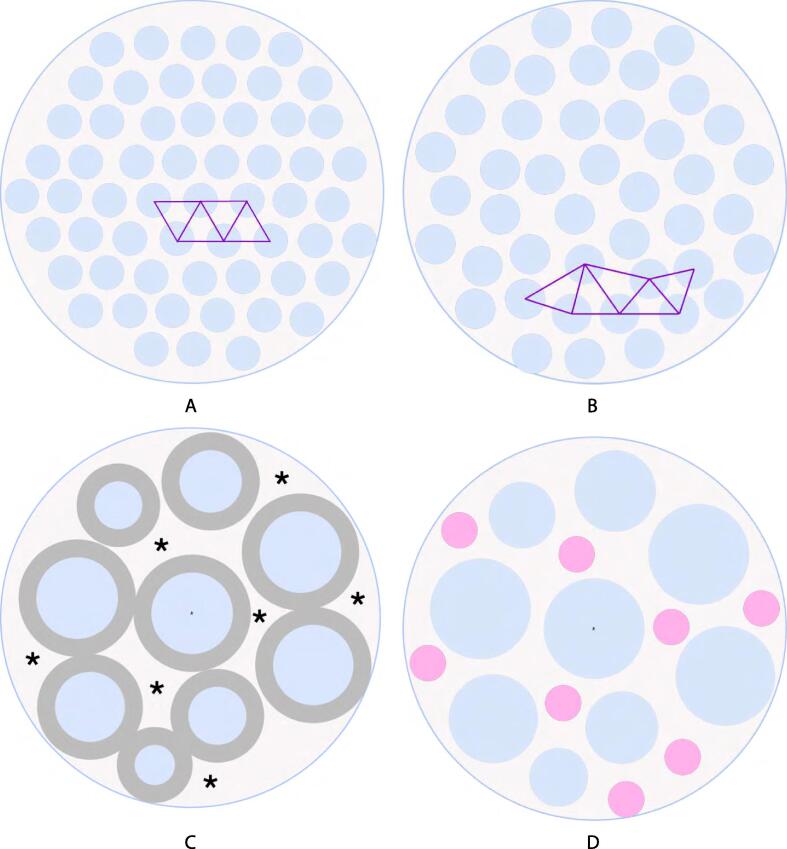
Our proposed mechanism for tendon growth and fibril deposition in early development. Each section is intended to show a fixed unit area of tendon tissue, as the fibrils inside this area grow. (A) Embryonic tendon. Here, fibrils are close to hexagonally packed (as indicated by the centre-to-centre connections in purple) and small. (B) Neonatal tendon. When the animal is born fibrils are small and uniform in size, but the fibril population no longer exhibits significant hexagonal packing, as indicated by the centre-to-centre lines in purple. (C)-(D): We suggest that a halo of collagen-associated matrix molecules of a specific size adhered to the surface of the fibril (indicated in grey), enforce a lower limit on how close together these fibrils can be. This leaves spaces (indicated with asterisks) for small fibrils (shown in pink) to be deposited or carved off from larger fibrils. We suggest this may establish the blueprint for the final fibril configuration as the FAF plateaus. We also suggest that fibrils continue to grow until tissue maturity has occurred by molecular accretion and by tip-to-tip fibril fusion.

This study examines the emergence of the complex fibril arrangement seen in adult tendon. Using a TEM time series in a murine model, we have presented a quantitative analysis of the evolution of various microstructural features. Our key findings give insight into the timing and character of the key transitions in fibril morphology and arrangement during development. We have illustrated some of our key findings in Fig. 13.

### 4.1 Hexagonal packing

The first of these findings concerns the arrangement of fibrils. Fibrils in embryo exhibit a hexagonal packing configuration. It has been reported that this hexagonal arrangement is stabilised in embryo with interconnections of a filamentous nature between the fibrils [8]. In this work, a hexagonal arrangement of fibrils is not seen at birth (Fig. 9B), nor are any of the stabilising filaments. This suggests that although the packing *appears* to be more regular at birth than in adulthood as the fibrils are of a similar size (see Table 2), the configuration is no closer to a hexagonal array at birth than at maturity (illustrated in Fig. 13 C-D). The transition from hexagonally arranged fibrils in embryo to the disordered arrangement of uniformly sized fibrils seen at birth could suggest a blueprint for the mature configuration, laid out by narrow fibrils which are to expand and fill the space between cells.

### 4.2 Multiple fibril populations

The tendon fibrils characterised in this study show near uniform diameter distribution at birth in agreement with previous work [8], [27], [5]. The characteristic multimodal diameter distribution (here measured using the MFD) is first visible as early as eight days after birth. By day 14, there are at least three populations of fibril diameters. Chang *et al*. [6], who studied 6–8 week-old mice, defined three fibril populations of fibrils, D1 fibrils have a diameter less than 75 nm, D2 fibrils are approximately 75–150 nm in diameter and D3 fibrils have a diameter greater than 150 nm. It is not straightforward to use this categorisation precisely with the early developmental data presented in this study, as the fibrils are immature. For example, two populations of fibrils can be seen on day 5 (Fig. 6), but all fibrils are below 75 nm in size. Whether these two populations are immature D1 fibrils, or an immature mix of D1, D2 and D3 fibrils is unclear. The mean MFD of these different populations increases with age (Fig. 6), though a persistent population of narrow fibrils (D1 fibrils in [6]) is seen throughout development and into adulthood. Deciding the range of diameters that defines fibrils as being D1, D2 or D3, is a matter of judgement, and indeed, it could be argued that there are more than three modes at later time points in Fig. 5. However, it is notable that a trimodal fit to the KDE gives an $R^{2}$-value greater than 0.98 at all time points (Fig. 6C). Therefore, we favour defining three populations over the complexity that would be introduced by considering four or more. We also note that the range of fibril MFDs increases with age (Fig. 4B), and that there is also a corresponding increase in FAF which eventually plateaus around 70% (Fig. 7A). The increased diversity in fibril size could directly contribute to increasing the FAF, with narrower fibrils being able to fill the gaps between larger fibrils. Eventually, the number of gaps that can be filled diminishes, leading to the plateau in the FAF.

### 4.3 The role of narrow (<100 nm in diameter) fibrils

The role of narrow (<100 nm in diameter) fibrils in adult tendon is unknown, but determining how exactly they relate to the narrow fibrils seen at birth may give insight into the purpose and regulation of the multimodal fibril population seen at maturity. The existence of narrow fibrils in postnatal tendon can be explained in one of three ways: they are present at birth and simply do not grow as large as other fibrils, they are synthesised *de novo* in early postnatal development, or they calve from larger fibrils once formed. There are a few suggestions in these results which can help to answer these questions about fibril growth.

Interestingly, fibripositors are visible in the tendon samples imaged in this work as late as day 25 (see Fig. 12), though they are rare. If narrow fibrils are being synthesised *de novo*, there would need to be large numbers of fibripositors to maintain their numbers, as the original fibrils are growing rapidly. The appearance of narrow collagen fibrils in the adult multimodal fibril population is, therefore, unlikely driven by deposition from fibripositors. It is worth noting that the fibrils inside the fibripositors recorded in this work at later time points are thicker (∼100 nm) than the 50 nm fibrils in the embryonic fibripositors (Fig. 12). These could be narrow fibrils that get stuck and erroneously grow inside a fibripositor structure, or fibrils which serve an altogether different function than those seen in early development.

Kalson *et al*. [5] report that there is the same number of fibrils per bundle (the space created by cell–cell contacts) at birth as there is at age 6 weeks. The results presented here do not dramatically contradict this finding as the relative fibril number at day 2 was found to be similar to that at day 56 in our data. However, our data suggest that new fibrils may be synthesised in the first week of development (as evidenced by the positive gradient in the first 7 days in Fig. 6). The drop in relative fibril number after the peak around day 10 suggests that either a significant number of fibrils is vanishing (which is unlikely), or that fibrils are fusing together. The data suggest that the fibrils are growing in size at a rate faster than the tissue until a plateau is reached, after which the fibrils and the tissue grow at the same rate. This is consistent with the observation that the FAF plateaus at around day 28. Three distinct populations of fibrils appear (Fig. 6B) at around day 14. From these results, it can be inferred that the most rapid microstructural reconfiguration and fibril expansion occurs within the first three weeks postnatal.

The images shown here do suggest fibrils exhibit a less circular profile as they grow (see Fig. 11). Though the decrease in circularity is small, this effect could give credence to the idea that the narrower fibrils seen in adult tendons are splitting from larger ones. Parry and Craig [28] suggested that adult fibrils have diameters which are multiples of 80 Å, which they interpreted as evidence of the existence of microfibrils, which join together laterally, leading to this stepped increase in fibril diameters. The prospective existence of the microfibril as a hypothesis for matrix expansion in tendons is certainly attractive. Smaller fibril fragments or microfibrils may be present in the tendons imaged in this work, though protein structures of this size will not be detectable using this kind of TEM. The resolution limit of this kind of TEM is around 5 nm, though objects of this size can be difficult to detect as they do not carry enough charge via the staining molecule to be recorded on the image plate. This means that objects much smaller than 20 nm in cross-section are not easily seen or distinguished, but being able to measure their numbers could give a clearer understanding of how and why the narrower fibril population is maintained throughout life, and why all the fibrils do not merge into one large mass.

### 4.4 Diameter regulation

Tendons are often subject to high forces and repeated loading. The purpose of the narrow fibrils is not known for certain, but it is possible that they are transitory and sacrificial, being turned over as part of the circadian cycle as they are damaged [6]. Another possibility is that their larger aspect ratios give them greater flexibility than the thicker fibrils. Tail tendons need to be more flexible than many other tendons, and it would be interesting to investigate whether similar sized populations of narrow fibrils are found in other tendons, such as the Achilles, for example. Some fibril growth is driven by accretion of collagen secreted from the cell (which was observed to be under circadian control [6]). It is noted that the fibrils closest to the cell are not noticeably larger than fibrils further from the cell [5], meaning that accretion of collagen molecules or microfibrils/fibril fragments cannot explain fibril growth on its own, assuming low cellular motility. Precise regulation of fibril diameter necessitates mechanistic control of molecular accretion, the key to which may lie in the way fibrils are packed. In this work, the width of the spaces between fibrils is observed to be constant throughout the time series (Fig. 9C). This may suggest that there is a halo of collagen-associated matrix molecules of a specific size adhered to the surface of the fibril, giving a lower limit on how close together these fibrils can be. If the protein content is insufficiently high, these molecules will not collect stain molecules and therefore will not be detectable by electron microscopy. If a fixed size gap between neighbouring molecules limits their proximity, the tendon can be better packed with fibrils if there are narrower ones which fill the spaces between the larger fibrils. It may be this which is the limiting factor on the narrow fibrils’ diameter: narrow fibrils fill the gaps between neighbouring large fibrils (indicated in Fig. 13 C-D). This would be to maximise the fibril area fraction, increase the collagen content of the tissue and provide the high tensile strength needed to transmit the loads seen in mammalian tendons.

### 4.5 The limitations of two-dimensional analysis

In this study, we have used two-dimensional, cross-sectional images as the basis of our analysis. This has allowed us to measure quantities such as fibril diameter, circularity and centre-to-centre distances, but it did not allow us to measure the evolution of three-dimensional features such as the fibril crimp distribution. In a recent paper, we developed an algorithm for automated tracking of fibrils in three dimensions, using serial block face-scanning electron microscopy data as the input [29]. Whilst collecting such data for the number of time points that we have considered in this study would be expensive, it would be valuable in the future to do so to allow for such analysis.

### 4.6 Future work – the potential of machine learning to enhance image analysis

We have analysed of a series of TEM images using traditional image segmentation and classification algorithms integrated into Cellprofiler [23]. Cellprofiler is based on the Python programming language, and uses the open-source and well-maintained scikit-image [30] and SciPy [31] libraries. This was done as these libraries are widely used, and it is straightforward to process large numbers of images with the CellProfiler package.

The tasks performed in this study could potentially also be conducted using machine learning (ML) techniques. There has been much interest in ML for both data analysis and image segmentation. Tools such as Ilastik [32] have made ML-based image analysis accessible to the scientific imaging community for some time. Deep learning and, in particular, convolutional neural networks (CNNs) have been used in a range of applications for biological imaging research. These include the segmentation of: cells imaged via light microscopy [33], sub-cellular structures such as mitochondria [34], and collagen fibres in the ECM [35]. ML performs well on classification tasks in this field. Pham *et al*. (2021) [36] used a CNN to classify histological sections as normal or scar tissue, and Shen *et al.* (2021) used CNNs, residual neural networks, and transfer learning to classify and predict mechanical states of cortical and trabecular bone tissue [37].

The use of ML has rapidly accelerated in the last few years due to the prevalence of new generative architectures such as generative adversarial networks (GANs) [38], transformers [39], and large language models (LLMs). Generative models can also be leveraged to solve problems in biomedical imaging and image segmentation. Vision transformers are now able to complete many image processing tasks that CNNs are traditionally used for [40]. Park *et al*. (2023) [41] used GANs to create synthetic data to complement the limited availability of training data for a model that aimed to quantify collagen fibres’ topological properties in microscopy-based collagen images from pathological tissue samples. GPT-4 (an LLM) was used to develop a vision-language conversational assistant for biomedical images using a large-scale biomedical figure-caption dataset from PubMed [42].

With these developments in mind, ML would be a natural next step to explore. It would be interesting to explore traditional ML segmentation methods to see how they compare with those used here, both in terms of the quality of segmentation and the speed of throughput. Classification and clustering algorithms could be employed to distinguish between multiple fibril populations as an extension to the statistical methods used in this work. LLMs, especially, offer intriguing opportunities for aggregating historical study images for meta-analysis. For instance, through the implementation of joint vision and language models, as demonstrated in [42], images of collagen fibrils from diverse research sources can be efficiently interpreted and described with scientific language. This tailored LLM enables swift parsing of extensive volumes of published material. Consequently, the rapid and automated examination of existing data facilitates novel hypotheses and the identification of new connections. It is important to highlight that the training of these models comes with considerable costs and challenges. However, if the endeavour proves successful, it has the potential to serve as an effective tool for acquiring additional evidence pertaining to the developmental mechanisms of collagen fibril growth — an inquiry that has occupied collagen researchers for many decades.

## 5 Conclusion

This study has indicated that many of the structural changes which occur postnatally take place within the first 14 days postnatal. This is evident in the plateauing of the FAF and the emergence of at least three fibril populations around this time. We have also observed that tendon fibrils are no longer arranged hexagonally once the animal is born and that the gaps between fibrils remain constant throughout development. This could indicate a mechanism for fibril growth involving surface-associated molecules limiting the proximity of adult collagen fibrils, which would suggest that the narrow fibril population which is seen in adulthood maintains the fibril area fraction by filling the spaces between larger fibrils necessitated by the minimum gap size. There is much left to be discovered about fibril growth in tendon, the mechanism regulating the fibril architecture and its effect on tendon mechanical behaviour.

## 6 Research Data

All original EM data and corresponding CellProfiler pipelines can be downloaded from DOI:10.48420/20161247. All subsequent analyses using the measured quantities were carried out using Python 3.9 with the following libraries: SciPy [31], SciKit-Image [30], alphashape pypi.org/project/alphashape and Shapely pypi.org/project/Shapely. The script used for the analysis can be found at github.com/hrh-uom/dev-ser-repo.

## Declaration of Competing Interest

The authors declare that they have no known competing financial interests or personal relationships that could have appeared to influence the work reported in this paper.

## Data Availability

Data will be made available on request.
