# Corrigendum to “A preliminary study into the emergence of tendon microstructure during postnatal development” [Matrix Biol. Plus 21 (2024) 100142]

**DOI:** 10.1016/j.mbplus.2024.100147

**Published:** 2024-05-17

**Authors:** Helena Raymond-Hayling, Yinhui Lu, Richa Garva, Tom Shearer, Karl E. Kadler

**Affiliations:** a Wellcome Centre for Cell Matrix Research, University of Manchester, United Kingdom; b Department of Mathematics, University of Manchester, United Kingdom; c Department of Materials, University of Manchester, United Kingdom

The authors regret that Richa Garva was inadvertently left off the author list of this publication. This was an unintentional oversight. The authors would like to apologise for any inconvenience caused and would like the author listing corrected as above.

All authors agree to this corrigendum.

Richa Garva agrees with the content and conclusions of the publication and with her name being added to the list of authors and with the position of her name in the listing.

